# The use of tear ferning test in cats for evaluation of ocular surface

**DOI:** 10.1186/s13028-020-00523-5

**Published:** 2020-05-26

**Authors:** Jéssica Fontes Veloso, Arianne Pontes Oriá, Ana Cláudia Santos Raposo, Ariane Jesus Lacerda, Cláudia Vital Borges Silva, Larissa Ferreira Lima, Renata Santiago Alberto Carlos

**Affiliations:** 1 Department of Veterinary Medicine, Federal University of Western Bahia, Avenida 23 de Agosto, Barra, Bahia 47100-000 Brazil; 2 School of Veterinary Medicine and Animal Science, Federal University of Bahia, Avenida Adhemar de Barros, Salvador, Bahia 40170-110 Brazil; 3 Department of Agrarian and Environmental Sciences, Santa Cruz State University, Rodovia Jorge Amado, Ilheus, Bahia 45662-900 Brazil

**Keywords:** Ferning pattern, Ocular surface disease, Tear film, Qualitative test

## Abstract

**Background:**

The tear film is a trilaminar fluid composed mainly of lipids, electrolytes, proteins and water. It is responsible for lubrication, nutrition and protection against microbial and toxic agents. Disruption of any these components may weaken the ocular surface, making it more susceptible to disease. Increasing evidence suggests that qualitative tear film deficiencies are an important predisposing factor or cause of some of the most common and challenging ocular diseases in cats, including conjunctivitis, corneal ulcer, spontaneous chronic corneal epithelial defects (SCCED), pigmentary keratitis, corneal sequestrum and dry eye syndrome. The aim of this study was to describe the tear ferning test in healthy cats and to compare the results by using two grading scales for humans. Tear samples were collected using Schirmer tear test (STT) strips from 60 healthy cats, and, after centrifuging the strips to obtain the samples, the aliquot was placed on clean microscope glass until it dried and the tear ferning patterns were observed under a polarized light microscope and classified according to the Rolando and Masmali grading scales.

**Results:**

Ferning patterns in the lower grades showed full crystallization with high density, without gaps between the ferns and branches, forming several nuclei that were easily distinguished. According to the Rolando scale, 50% (60/120), 46.6% (56/120) and 3.4% (4/120) of eyes showed type I, II and III patterns, respectively. According to the Masmali scale, 15% (18/120), 56.6% (68/120 eyes) and 28.4% (34/120) of eyes showed grade 0, 1 and 2 patterns, respectively. No difference was observed between the right and left eyes for both Rolando (P = 0.225) and Masmali (P = 0.683) scales.

**Conclusions:**

The tear ferning test is a qualitative test that can be used in cats as a complementary evaluation of the ocular surface. While the Rolando scale showed an increased prevalence of types I and II, the Masmali scale showed an increased prevalence of grades 1 and 2. This can be attributed to the species-specific differences between human and feline tear film. So Masmali grade 2 can be considered a normal tear pattern for the species, because all the cats used in study were clinically healthy. For this reason, future complementary studies are necessary for comparing healthy eyes and eyes with different ocular surface disease in cats. Both scales can be feasible options for grading tear crystallization in cats, but as Rolando scale included 96.6% of the samples in the 2 types that are considered normal for humans, we think that this scale seemed to be more precise to classify crystallization pattern in cats. The crystallization patterns observed in this study can form the basis for standardizing ocular surface parameters in cats.

## Background

Tear film (TF) is a viscous and complex trilaminar fluid composed mainly of lipids, electrolytes, proteins and water [1]. Normal TF dynamics require adequate tear production, tear retention on the ocular surface and balanced tear drainage. The TF is responsible for lubrication, nutrition and protection against microbial and toxic agents [2]. Disruption of TF dynamics can lead to dry eye or the ocular surface can become more susceptible to the onset of diseases [3–5].

Different tools can be used to evaluate the ocular surface [6]. Schirmer tear test-1 (STT-1) is considered the gold standard method for measuring tear production; however, it does not measure tear quality [7]. The tear ferning test (TFT) is a qualitative test developed for humans, and it has become a useful diagnostic tool in tear ferning research. Various ferning patterns can be observed as a result of tear crystallization after evaporation of lacrimal samples, and these patterns depend mostly on TF composition [8]. In addition, tear crystallization may be affected by humidity and temperature [9, 10]. Therefore, changes in ferning patterns are believed to reflect possible changes in both composition and stability of the TF [10].

In humans, Rolando suggested the first grading scale for the TFT, wherein types I and II indicated normal TFs, while types III and IV indicated abnormal TFs [9]. Thereafter, Masmali developed a TFT grading scale for humans, with the aim of addressing the gaps in previous classification systems. According to this scale, TFs with grades 0 and 1 were considered normal, while those with grades 2, 3 and 4 were considered abnormal [10].

In addition to humans [6, 10, 11], the Rolando and Masmali grading scales have been applied in horses [12], dogs [13, 14], camels [11] and capuchin monkeys [15]. Although no record in the veterinary medicine literature documents the use of a grading scale specifically for animals, previous studies have successfully used the human grading scales in some animal species, thus showing that the TFT is a feasible and complementary test that is simple and inexpensive for ocular surface assessment [12, 13].

Increasing evidence suggests that qualitative TF deficiencies are an important cofactor or cause of some of the most common and challenging ocular diseases in cats, including conjunctivitis, corneal ulcer, spontaneous chronic corneal epithelial defects (SCCED), pigmentary keratitis, corneal sequestrum and dry eye syndrome [16–21]. Despite this, the TFT has not yet been performed in cats, even though it is already considered a complementary diagnostic tool in ocular surface research. Therefore, this lack of applied research on tear ferning in cats justifies an investigation of the application of the two TFT grading scales in healthy cats to understand the potential role of tear deficiency and the value of tear testing in cats.

## Methods

### Study population

The study included 60 mixed breed cats (120 eyes) of which 33 were females (55.0%) and 27 were males (45.0%), aged between 1 and 8 years (mean 2.5 ± 1.86 years), with no complaint of illness. Additionally, they had to be vaccinated, without systemic or ocular signs of disease, without any history of ocular secretion or injury and with STT-1 values within the normal range for the species. The animals also had normal, complete blood counts and biochemical test results, including those for urea, creatinine, alanine aminotransferase and alkaline phosphatase. Moreover, all animals tested negative for feline coronavirus, feline leukemia virus and feline immunodeficiency virus.

Before data collection, the STT-1 (Schirmer Tear Test; Ophthalmos, São Paulo, Brazil) was performed and used as a screening method to measure tear production. The median value and interquartile range (median ± S-IQR) was 20 ± 7 mm/min, and the 95%-confidence intervals (CI) was between 18.4 and 19.8 mm/min.

Evaluations of the ocular adnexa and anterior segment were performed using a slit-lamp biomicroscope (Vision Class II BL IIIB/YZ30T; Ramos Mejia, São Paulo, Brazil). Intraocular pressure (IOP) was measured using a rebound tonometer (Icare tonometer; Icare Finland Oy, Vantaa, Finland); For IOP the median ± S-IQR value was 22 ± 6 mmHg, and the CI was between 22.3 and 24.1 mmHg. The ocular surface was evaluated using fluorescein dyes (Fluorescein test; Ophthalmos), TF breakup time (TFBT) (range 5–9 s) and lissamine green (Lissamine green test; Ophthalmos). The IOP, fluorescein, TFBT and lissamine green tests were performed after tear sampling to avoid any interference with tear crystallization. All data were collected in a room in which the temperature and humidity were controlled.

This study was approved by the Ethics Committee on Animal Experimentation of the Use of Animals from the State University of Santa Cruz, Brazil (protocol no. 003/17). All procedures were conducted in accordance with the Association for Research in Vision and Ophthalmology’s (ARVO) Statement for the Use of Animals in Ophthalmic and Vision Research and NIH statement.

### Sample collection and TFT

Tear samples were collected between 8:00 a.m. and 11:30 a.m., first from the right eye and then the left eye. Once the tear wetted 30 mm on the Schirmer strips, which were the same ones used for STT-1, the strips were immediately placed in a 0.5 ml microtube (Protein LoBind Tubes; Eppendorf, São Paulo, Brazil) and conditioned in a thermal box until centrifugation. Immediately before centrifugation, the bottom of the 0.5 mL microtube was punctured and it was inserted into a larger 2.0 mL microcentrifuge tube (Protein LoBind Tubes; Eppendorf) for extracting the tear fluid as previously described by Oria et al. [13]. The tear fluid was obtained through centrifugation (25.830 *g* for 10 min at 4 °C) of the Schirmer strips (Schirmer Tear Test; Ophthalmos).

During sample collection and processing, the temperature and humidity ranged from 20.9 to 27.1 °C and 42 to 62%, respectively. A teardrop of approximately 2 µL was deposited on a glass slide, at the center of a circle made previously and the tear ferning time (i.e., from tear deposition until drying) was measured using a digital timer. After complete drying, the slides were evaluated under a 10× lens magnification polarized light microscope with a camera (Microscope Scope A.1/AX10 Axion Cam ICc5; Zeiss, São Paulo, Brazil). The acquired images were classified and the formation of branches, angulations and zones of transitions were evaluated according to the proposed scales of Rolando et al. [9] and Masmali et al. [10].

The images of the ferning patterns were classified by three separate blinded examiners (APO, ACSR and AJL) from the Veterinary Ophthalmology Research Group of the School of Veterinary Medicine and Zootechny of Federal University of Bahia. All the examiners had experience of and expertise in the use of the scales. The final grading was assigned on the basis of agreement between the classifications of at least two of the three examiners. Tear ferning patterns were classified according to the Rolando grading scale (types I, II, III and IV) and according to the Masmali grading scale (grades 0, 1, 2, 3 and 4).

### Statistical analysis

Statistical analysis was conducted using IBM SPSS Statistics for Windows, Version 22.0 (IBM Corp.), and the R Software for Windows, Version 3.6.1 with package irr was used for Cohen’s kappa coefficient (k). The level of significance was set at 5% (P < 0.05) and CI at 95%. The Shapiro–Wilk test was used to test data normality of the TFT values. Wilcoxon test was used for comparison of the same variables between eyes on each scale. The Mann–Whitney test was used to compare classification (both scales) with sex. Age was correlated with the classifications obtained through the Spearman test. Cohen’s kappa coefficient was used to verify the agreement among the examiners in each scale.

## Results

Crystallization occurred in the tear samples of all animals submitted to the test, with an average time of 14.6 ± 4.3 min. The crystallization patterns that received lower grades showed full crystallization with high density, without gaps between the ferns and branches, forming several nuclei that were easily distinguished. The branches showed medium length and were thin, with well-defined primary and secondary ramifications. As the grade increased, the nuclei lost definition, gaps were observed between the branches and, sometimes, coarse crystals were formed. Nevertheless, crystallization was observed even in the higher grades.

**Fig. 1:**
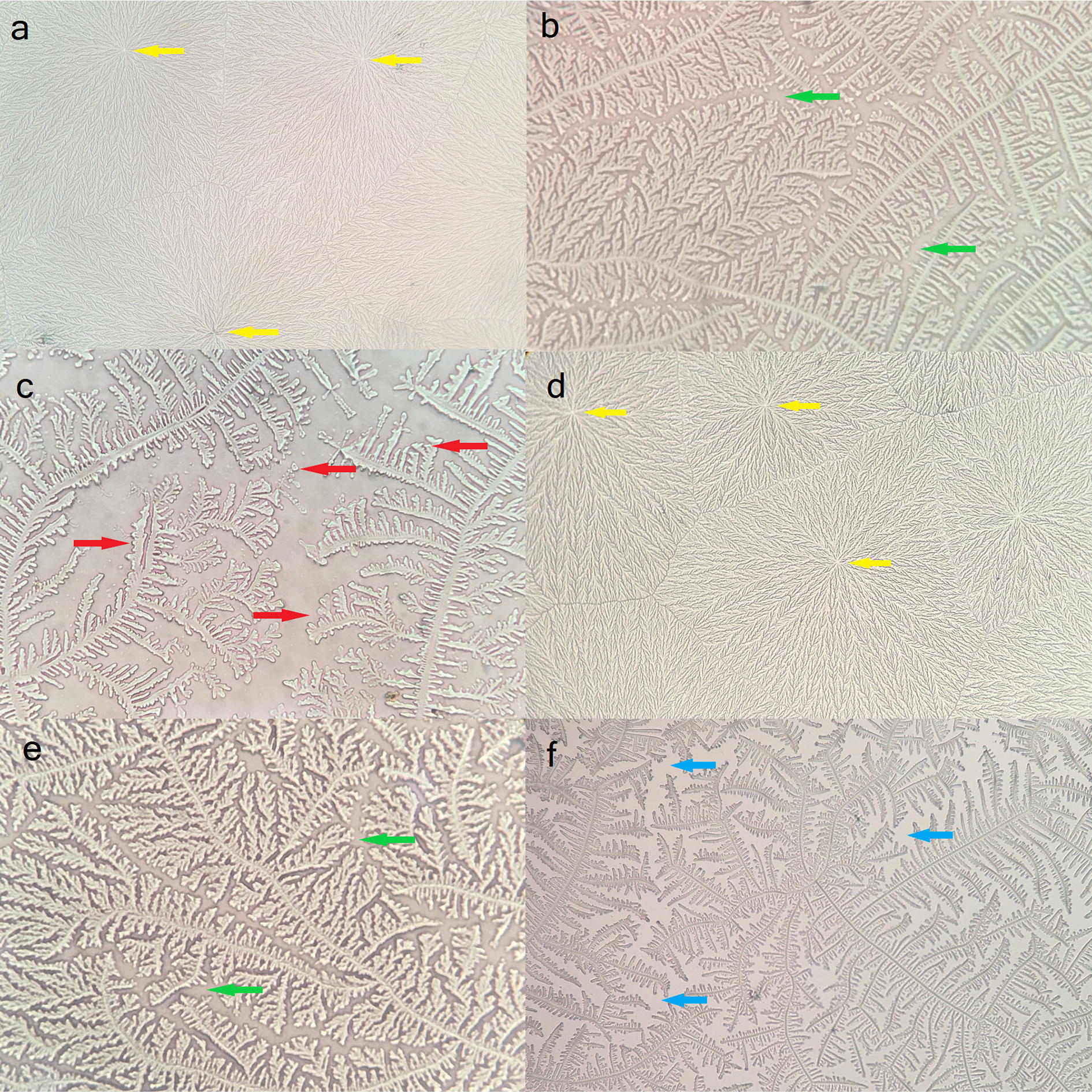
Examples of tear ferning patterns in cats according to (**a**–**c**) the Rolando and **d**–**f**) the Masmali grading scales. **a** Type I representation: dendritic fern growth is uniform, nuclei (*yellow arrows*) are easily distinguished and no gaps are seen between the branches. **b** Type II: small spaces (*green arrows*) begin to appear between the branches and the ferns are thicker. **c** Type III: incomplete crystallization process; coarse crystals (*red arrows*) are formed in single and small size, and branches are rare. **d** Grade 0 representation: full crystallization without gaps between the ferns and branches, and nuclei well demarcated. **e** Grade 1: branch density is decreased and small spaces appear between them. **f** Grade 2: small branches—sometimes thick and large—with clear gaps between the ferns *(blue arrows*), and nuclei not visible. Note the similarity between panel (**a**) and (**d**); between (**b**) and (**e**); Furthermore panel (**b**) is closer to (**e**), whereas (**b**) and (**f**) appear more similar than (**c**) and (**f**), suggesting that Roland type I is similar to Masmali grade 0, and Rolando type II shares some common features with both Masmali grades 1 and 2

As described in methodology, after the ferning, the images were classified by resemblance to Rolando scale (Fig. 1a–c) and Masmali scale (Fig. 1d–f). The results obtained for each grading scale are expressed in Table 1.

**Table 1:** Grading results for Rolando scale and Masmali scale for tear ferning in healthy cats

Scale	Classification	Results
Rolando	I	60 (50%)
	II	56 (46.6%)
	III	4 (3.4%)
	IV	0
		120 (100%)
Masmali	0	18 (15%)
	1	68 (56.6%)
	2	34 (28.4%)
	3	0
	4	0
		120 (100%)

The obtained classifications did not show a normal distribution (P < 0.001). The Wilcoxon test revealed no difference between the right and left eyes for both Rolando (P = 0.225) and Masmali (P = 0.683) scales. The median ± S-IQR value for the Rolando scale was 2 ± 0.5 and the CI was between 1.47 and 1.77. For the Masmali scale, the median ± S-IQR value was 1 ± 0.5 and the CI was between 1.0 and 1.62. No differences were found in relation to sex and there was no correlation between the scores obtained and the age of the study animals. The Kappa coefficient of agreement obtained for each pair of examiners for the Rolando and Masmali scales are presented in Table 2. All concordances were significant (P < 0.05), except for the comparison between examiner A and B for the Rolando scale. The examiners B and C had strong agreement in their classifications of both scales.

**Table 2:** Cohen’s kappa agreement coefficient and P value among the examiners (A, B and C) for tear crystallization of cats according to Rolando and Masmali scales

Examiner	Examiner	Rolando scale		Masmali scale	
		*k*	P-value	*k*	P-value
A	B	0.038^a^	0.615^*^	0.141^b^	0.038
A	C	0.17^b^	0.018	0.199^b^	0.004
B	C	0.537^c^	< 0.001	0.617^d^	0

^a^ poor correlation; ^b^ slight correlation; ^c^ moderate correlation; ^d^ strong correlation; ^*^ P > 0.05, therefore not significant

## Discussion

Qualitative test of the TF is of great importance in animals that are affected by ocular surface disease and is complementary to quantitative assessment of tear production, as the latter does not guarantee a TF of good quality [20]. In this study, we have shown that the TFT can be easily and safely performed in cats and has promising potential to be considered in the future as a valuable research tool in veterinary ophthalmology, as already reported by other authors [11–14]. Nevertheless, no other study to date has performed the TFT in cats.

STT strips were used to collect the tear samples for the TFT, because previous studies have shown that these strips minimize conjunctival lesions that may modify the crystallization pattern; moreover, studies recommend that the tear collection technique used in cats should be minimally invasive [13, 22]. Furthermore, this technique can be used without necessitating further manipulation of the eye. The STT strip is made of soft, absorbent and malleable paper, thereby minimizing reflex tearing or damage to the cornea and adjacent structures during tear collection. In contrast, tear collection with capillary microtube can lead to eye damage caused by voluntary and involuntary movements of cats, as they were not sedated in this study [5, 22, 23]. This is especially important if more than one test is performed during the same examination, as the manipulation of cats’ eyes can lead to exfoliation of conjunctival cells and/or release of TF components that are not normally expressed. Therefore, STT-1 tear sample collection was performed before any other clinical and ophthalmic tests [24].

We found differences when comparing feline TF samples to the descriptions of TF made for other species. The differences observed included branching pattern density when compared to humans [6, 10, 11], dogs [13, 14], capuchin monkeys [15] and horses [12]; crystals arrangement when confronting with descriptions of horses [12], capuchin monkeys [15] and dogs [13, 14]; and nuclei visibility when compared to all species previously mentioned [6, 10–15] plus camels [11]. All these pattern differences can be attributed to variations in tear composition [10], sampling method [25], temperature and humidity [26].

Types I and II of the Rolando scale were observed in 96.6% of the evaluated animals, and grades 0 and 1 of the Masmali scale were observed in 71.6%; these findings were similar to those of previous studies on other animals and healthy humans [10–13, 27]. However, the pattern with the second highest frequency the Masmali scale (28.4% in grade 2) is considered unhealthy in humans [10]. Raposo et al. [15] attributed the high frequency of Masmali grade 2 (72.7%) in lacrimal film of capuchin monkeys samples to species-specific characteristics, since all the animals were also healthy. Under these circumstances, the occurrence of grade 2 patterns in the cats evaluated in the present study is likely to be a standard of normality for cats, since the animals were clinically healthy, and no statistical difference was observed regarding gender or age.

Another explanation for the TF patterns observed in this study could be the osmolarity of feline tears, which is higher than that of human tears. Electrolyte concentration has already been reported as a cause of changes in crystallization patterns [14, 20, 28]. Although tear osmolarity was not assessed in this study, it could be a possible explanation for the observed patterns, and hence, more research is warranted on this aspect.

The lack of strong agreement among examiners suggests that observation of crystal’s morphology details is observer-dependent and so indicates that this assessment requires training and harmonization between different examiners. Therefore, the adoption of the two scales aimed to minimize the subjectivity of the test in cat samples. A similar observation has already been made in TFT studies for other species, such as dogs [13] and capuchin monkeys [15], and as happened in these studies we suggest that the adoption of a species-specific scale can be a way to enhance pattern classification. It is worth noting that the TFT is described as a complementary test to other methods [9, 10, 20, 25, 26], and will certainly contribute to better understanding of the ocular surface in all species.

Aqueous tear deficiency (“Dry Eye Syndrome”) is seldom reported in cats [21], and none of the cats in this study showed signs of ocular disease, including corneal surface disease. Therefore, these findings suggest that Rolando types I and II and Masmali grades 0, 1 and 2 reflect normal TFT results in cats. While the results of most cats fell within types I and II of Rolando scale the grades 1 and 2 of Masmali scale, this can be attributed to the species-specific differences between human and feline tear film. Masmali grade 2 can be considered a normal tear pattern for the species, because cats studied were clinically healthy. For this reason, future complementary studies are necessary in comparing healthy eyes with different ocular surface disease in cats. Both scales can be feasible options for grading tear crystallization in cats, but as Rolando scale included 96.6% of the samples in the 2 types that are considered normal for humans, we think that this scale seemed to be more precise to classify crystallization pattern in cats. The crystallization patterns observed in this study can form the basis for standardizing the TFT of domestic cats.

## Conclusions

The TFT is a feasible, inexpensive and low-risk test in cats. Our findings revealed high prevalence of the type I pattern according to the Rolando scale and of the grade 1 and 2 pattern according to the Masmali scale. These findings suggest that the TFT can be used as a complementary test for evaluating the ocular surface of cats and can provide insights into qualitative deficiencies of TF. We believe these findings will enhance our understanding of qualitative tear film disease and aid the design of prospective, case-controlled studies to better define the TFT results in various feline ocular diseases, such as conjunctivitis, corneal ulcer, SCCED, pigmentary keratitis, corneal sequestrum and dry eye syndrome.

## Data Availability

The datasets used and/or analysed during the current study are available from the corresponding author on reasonable request.

## Prior publication

Data have not been published previously.
